# Nanosponge-Based Composite Gel Polymer Electrolyte for Safer Li-O_2_ Batteries

**DOI:** 10.3390/polym13101625

**Published:** 2021-05-17

**Authors:** Julia Amici, Claudia Torchio, Daniele Versaci, Davide Dessantis, Andrea Marchisio, Fabrizio Caldera, Federico Bella, Carlotta Francia, Silvia Bodoardo

**Affiliations:** 1 Electrochemistry Group, Department of Applied Science and Technology, Politecnico di Torino, C.so D.ca degli Abruzzi 24, 10128 Torino, Italy; claudia.torchio@polito.it (C.T.); daniele.versaci@polito.it (D.V.); davide.dessantis@polito.it (D.D.); andrea.marchisio@polito.it (A.M.); federico.bella@polito.it (F.B.); carlotta.francia@polito.it (C.F.); silvia.bodoardo@polito.it (S.B.); 2 Department of Chemistry, Università degli Studi di Torino, Via Pietro Giuria 7, 10125 Torino, Italy; fabrizio.caldera@unito.it

**Keywords:** Li-O_2_ cell, composite gel polymer electrolyte, nanosponge, O_2_ cross-over

## Abstract

Li-O_2_ batteries represent a promising rechargeable battery candidate to answer the energy challenges our world is facing, thanks to their ultrahigh theoretical energy density. However, the poor cycling stability of the Li-O_2_ system and, overall, important safety issues due to the formation of Li dendrites, combined with the use of organic liquid electrolytes and O_2_ cross-over, inhibit their practical applications. As a solution to these various issues, we propose a composite gel polymer electrolyte consisting of a highly cross-linked polymer matrix, containing a dextrin-based nanosponge and activated with a liquid electrolyte. The polymer matrix, easily obtained by thermally activated one pot free radical polymerization in bulk, allows to limit dendrite nucleation and growth thanks to its cross-linked structure. At the same time, the nanosponge limits the O_2_ cross-over and avoids the formation of crystalline domains in the polymer matrix, which, combined with the liquid electrolyte, allows a good ionic conductivity at room temperature. Such a composite gel polymer electrolyte, tested in a cell containing Li metal as anode and a simple commercial gas diffusion layer, without any catalyst, as cathode demonstrates a full capacity of 5.05 mAh cm^−2^ as well as improved reversibility upon cycling, compared to a cell containing liquid electrolyte.

## 1. Introduction

The ratio of energy production coming from renewable sources is constantly increasing and, by its aleatory nature, it requires efficient storage solutions. Another important factor to be considered is the explosion of the electric vehicles market, with a request for performances at least equivalent to the ones of fossil fueled vehicles. From these considerations, the need for batteries with more and more energy and power density arises. To this day, Li-ion batteries are the ones meeting the widest market and range of utilization [1]. However, this technology is reaching its theoretical values and it will not be able to sustain the challenges of future applications, mainly in terms of energy density. On the other hand, while far from the market, Li-O_2_ batteries represent a valid alternative by combining the low density and highly reactive metallic Li to the abundant, low-cost, and environmentally friendly O_2_, thus allowing to reach a theoretical specific energy as high as 3582 Wh kg^−1^ [2]. Proof of the reversibility of this system was demonstrated in the early 2000s using an organic liquid electrolyte and a MnO_2_-based catalyst [3]. Since then, many efforts have been made to further understand this chemistry and solve its issues to render it more stable and safer. Indeed, one of the drawbacks of this technology is linked to the use of metallic Li, which, upon battery recharge, tends to be inhomogeneously redeposited at the anode, thus forming so-called dendrites, 3D needles which can either break down creating “dead Li” and deplete the active material, thus causing capacity loss, or grow to pierce the separator and short-circuit the cell, leading to hazards of thermal runaway and even explosion accidents [4]. Another problematic issue is the O_2_ cross-over, meaning that the excess of unreacted O_2_ at the cathode dissolves in the liquid electrolyte and goes to react directly with metallic Li, causing the formation of a passivating film on the anode surface, once again hampering the cell safety [5]. Last but not least, in an ideal Li-O_2_ open battery, the use of traditional liquid electrolytes would cause additional issues, such as solvent evaporation, leakage, and flammability [4].

A common solution to these issues consists in the creation of a physical barrier blocking dendrite growth and penetration as well as O_2_ cross-over. Such barrier can either be a functionalized separator or a solid electrolyte [5,6,7]. The replacement of liquid electrolytes with solid ones does not change the fundamental reaction of the Li-O_2_ batteries, which is still the well-known formation and decomposition of Li_2_O_2_. However, only a few works about ceramic-based Li-O_2_ batteries have been reported because of the difficulty in establishing a tri-phase (O_2_/e^−^/Li^+^) reaction between the cathode and a ceramic-based electrolyte [8,9,10]. Moreover, solid state electrolytes with low conductivity at room temperature require an elevated operating temperature of the cells, which aggravates the side-reactions and the safety hazards [4]. Therefore, in the last few years, multifunctional polymer-based electrolytes for quasi-solid-state Li-O_2_ batteries have been thoroughly studied [8]. However, polymer electrolytes share a common issue with ceramic electrolytes, which is their high interfacial resistance and low ionic conductivity, both limiting their practical applications at ambient temperature [11,12]. On the other hand, gel polymer electrolytes (GPEs), composed of liquid electrolytes entrapped in polymer matrices, have been successfully used for Li-ion battery applications due to their low interfacial resistances and high ionic conductivity [11,13,14]. GPEs with different polymer-solvent combinations have been developed in Li-O_2_ batteries and they have been shown to efficiently protect Li anode from oxygen cross-over, as well as limit electrolyte evaporation [15,16,17]. Furthermore, the incorporation of fillers, organic or inorganic, to both polymer and liquid electrolytes has been shown to improve the Li^+^ transport properties, such as Li-ion transference number and ionic conductivity, mainly through their interactions with the polymer, solvent, or salt [11,18,19,20]. One particularly interesting organic filler is the dextrin-based nanosponge. Dextrin-based nanosponges (NS) are hyper-crosslinked polymers characterized by the ability to encapsulate a great variety of substances in the liquid phase. More importantly, dextrin-based NS are nanostructured within a three-dimensional network. Our group already reported the use of NS encapsulated in polymer matrices for different applications [21,22].

In this work, we report the preparation of a methacrylate-based polymer matrix by a solvent-free, thermally induced, radical polymerization, encompassing NS and swollen in liquid electrolyte in order to obtain a composite gel polymer electrolyte (CGPE). This simple process allowed us to obtain a highly cross-linked CGPE with good mechanical properties and numerous long polymer chains rich of ethoxy groups, favoring Li-ions conduction. Additionally, the integration of NS as an additive permits effectively reducing the O_2_ cross-over upon the electrolyte, thus stabilizing the metallic Li interface while increasing the O_2_ content at the cathode side and improving reactions kinetics, allowing better cyclability and greatly improving the cell safety.

## 2. Materials and Methods

### 2.1. Materials

Kleptose Linecaps 17 Lab 4118 (LC, Mw 12,000 Da) and β-cyclodextrin (β-CD) were provided by Roquette Frères (Lestrem, France). Sodium hypophosphite monohydrate, citric acid, and polyethylene glycol diacrylate as crosslinking agent (PEGDA575, Mn 575) were obtained from Merck, Darmstadt, Germany. Buthyl methacrylate (BMA, 99%) and benzoyl peroxide (BPO, 75%) were obtained from Acros Organics, Geel, Belgium. Lithium bis- (tri-fluoromethylsulfonyl) imide (LiTFSI) 0.5 M in dimethyl sulfoxide (DMSO) (battery grade) was provided by Solvionic (Toulouse, France). Unless specified differently, chemicals were used as received.

### 2.2. Dextrin-Based Nanosponges Preparation

The dextrin-cyclodextrin NS was prepared by solubilizing 21.00 g of LC, 2.10 g of β-CD, 5.00 g of sodium hypophosphite monohydrate, and 71.00 g of citric acid in 50 mL of deionized water, as reported elsewhere [23]. Citric acid was the cross-linking agent. The solution was placed in an oven (Memmert VO500, Schwabach, Germany) and heated for 100 h at 80 °C under low pressure (30 mbar). The obtained NS were grounded in mortar, washed with excess of deionized water, and rinsed with acetone through Buchner filtration (FESEM micrograph of the raw nanosponge reported on Figure S1).

### 2.3. CGPEs Preparation

**Figure 1:**
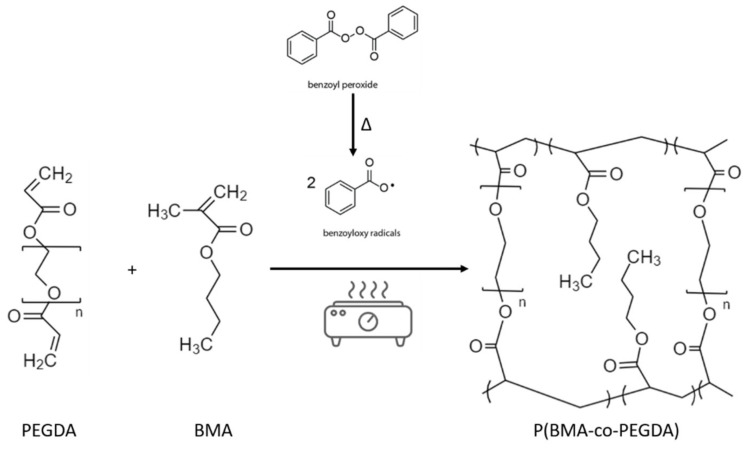
Schematic representation of the polymerization process.

The synthesis was performed by thermally induced, free radical polymerization, in bulk, as schematically represented in Figure 1, in a controlled Ar atmosphere glovebox (Mbraun Labstar, Stratham, NH, USA, O_2_ and H_2_O contents < 0.5 ppm) to avoid oxygen inhibition. The precursor solutions contained: BMA as monomer in various proportions, 10 wt% of PEGDA575 as crosslinking agent, 1 wt% (with respect to the polymer content) of BPO as thermo-initiator, and eventually NS as additive in different proportions. The solution was magnetically stirred and heated to 80 °C for 30 min before being casted on a glass-slide. Afterward, the temperature was set to 45 °C for 20 h and finally to 100 °C for 2 h. The prepared membranes were peeled from the glass slide and immersed in the liquid electrolyte (LiTFSI 0.5 M in DMSO) for 2 h for activation.

### 2.4. CGPEs Characterization

The CGPEs’ morphologies were examined using a field emission scanning electron microscope (FESEM, ZEISS Supra 40, Oberkochen, Germany). Thermal stability was assessed by thermogravimetric analysis (TGA) with a Mettler TGA/SDTA 851 instrument (Columbus, OH, USA), in air, between 25 and 800 °C at 10 °C min^−1^. The samples’ structure was studied by X-ray diffraction (XRD) analysis, carried out using a high resolution Philips X’pert MPD powder diffractometer (Philips, Amsterdam, The Netherlands), equipped with Cu Kα radiation (V = 40 kV, I = 30 mA) and a curved graphite secondary monochromator. The diffraction profiles were collected in the 2θ range between 15° and 90°, with an acquisition step of 0.018° and a time per step of 10 s using a solid state PIXcel-1D detector with 255 active channels. Oxygen permeability was measured by an Extrasolution MultiPerm instrument (Pieve Fosciana, Italy). The CGPE was mounted on the instrument with a surface reducing frame to realize an exposed surface of 2.27 cm^2^. Analyses were performed using a pressure of 1.0 atm, at 25.8 °C with a humidity level of 85%. The liquid electrolyte uptake (LEU) was obtained by measuring the weight of the dry membrane and the saturated membrane after immersion for 2 h in the electrolyte. The electrolyte uptake was calculated according to Equation (1):

LEU = ([M_e_ − M_0_]M_0_) × 100

where M_0_ and M_e_ are the weights of the membrane before and after immersion, respectively. The ionic conductivity of the membrane was determined by electrochemical impedance spectroscopy (EIS) in the frequency range between 100 kHz and 1 Hz at open circuit potential using a CHI potentiostat instrument (IJ Cambria Scientific Ltd., Llanelli, UK). Discs of 2.54 cm^2^ were cut from the different CGPEs swollen in the electrolyte for 2 h and sandwiched between two stainless steel blocking electrodes (ECC-Std test cells, EL-CELL GmbH, Hamburg, Germany). The assembled cells were kept in an oven and tested between 25 and 60 °C. The resistance of the electrolyte was given by the high-frequency intercept determined by analyzing the impedance response. The ionic conductivity was calculated at each temperature using Equation (2):

σ = (l/A) × (1/R_Ω_) (2)

where l is the membrane thickness, A is the membrane surface area, and R_Ω_ is the resistance value at the high-frequency intercept. The electrochemical stability was evaluated by linear sweep voltammetry (LSV) performed with a SS|CGPE|Li cell (ECC-Std) at a scan rate of 0.5 mV s^−1^ from 0 to 6 V vs. Li/Li^+^ at room-temperature. The Li-ion transference number (t_Li+_) was investigated by a potentiostatic polarization method using a symmetrical Li–Li cell (Li|CGPE|Li). The Li-ion transference number t_Li+_ can be calculated following Equation (3):

t_Li+_ = [I_s_ × (ΔV − I_0_ R_0_)]/[I_0_ × (ΔV − I_s_ R_s_)] (3)

where I_0_ and I_S_ are the initial and steady-state current values, respectively. ΔV is the applied DC potential (10 mV); R_0_ and R_S_ are the interfacial impedance values at initial and steady state, respectively. The effect of the membrane on Li plating and stripping was studied using a Li/Li symmetrical cell configuration, with the corresponding CGPE sandwiched in between (ECC-Std). A control-cell was assembled as follows: Li|Glass fiber separator + 1 M LiTFSI in DMSO|Li (ECC-Std). The current density and the related discharge capacity were 0.3 mA cm^−2^ and 0.3 mAh cm^−2^, 0.5 mA cm^−2^ and 0.5 mAh cm^−2^, 1 mA cm^−2^ and 1 mAh cm^−2^, respectively. For each characterization technique, the results were compared with the ones of a control-cell, assembled with a commercial glass fiber separator impregnated with 1 M LiTFSI in DMSO in place of the different CGPEs.

For full-cell testing, discs with an area of 2.54 cm^2^ were cut from a commercial carbon paper gas diffusion layer (GDL-24BC, SIGRACET SGL Technologies, Meitingen, Germany) dried in vacuum at 120 °C for 6 h and used as a cathode. A Li disc (18 mm × 0.2 mm, Chemetall s.r.l., Giussano, Italy) was used at the anode, while either a commercial disc of glass fiber (18 mm × 0.65 mm, ECC1-01-0012-A/L, EL-CELL, Hamburg, Germany) or the selected CGPE was used as the separator. A solution of LiTFSI 0.5 M in DMSO was the electrolyte. The amount of electrolyte was 200 µL in the cells with the glass fiber separator, while the different CGPEs were activated in the liquid electrolyte for 2 h. The cells were assembled in an Ar-filled glove box (Mbraun Labstar) using an ECC-Air electrochemical cell design (EL-Cell, GmbH). The cells were galvanostatically discharged and charged by an Arbin BT-2000 battery tester (College Station, TX, USA) at room temperature, between 2.25 and 4.4 V vs. Li/Li^+^ at 0.025 mA cm^−2^. During measurements, pure O_2_ at a flow rate of 3.0 mL min^−1^ was constantly fluxed. Prior to each test, cells rested under oxygen flow for 6 h at open-circuit voltage (OCV).

## 3. Results and Discussion

The P(BMA-co-PEGDA) membranes were synthesized by a radical copolymerization of BMA and PEGDA (see Figure 1), allowing to obtain a highly cross-linked polymer matrix. Both monomer and polymer were chosen first for their characteristics, in particular the presence of numerous ethoxy groups, playing a crucial role in Li-ions conduction and second, for their ability to be cross-linked together. Indeed, a cross-linked polymer matrix confers a more robust character to polymer electrolytes, thus helping reduce Li dendrite permeation and the associated safety hazards [24]. The as obtained membranes were successively activated in liquid electrolyte and renamed CGPE.

**Figure 2:**
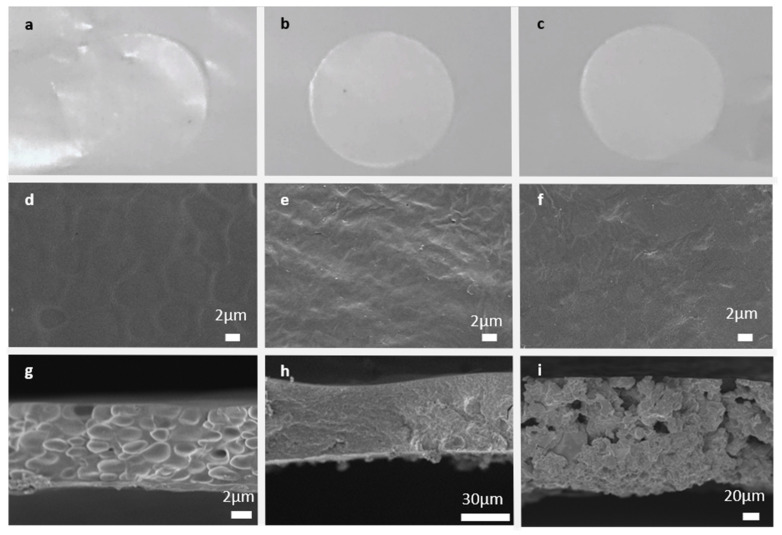
Pictures of the different CGPEs: without NS (**a**), with 5 wt% NS (**b**), with 10 wt% NS (**c**). FESEM micrographs of the surface of CGPE without NS (**d**), CGPE with 5 wt% NS (**e**), CGPE with 10 wt% NS (**f**). FESEM micrographs of the cross-section of CGPE without NS (**g**), CGPE with 5 wt% NS (**h**), CGPE with 10 wt% NS (**i**).

Pictures of the obtained CGPEs with and without NS are reported in Figure 2a–c. The CGPE without NS was perfectly transparent, while the other two were homogeneously white, demonstrating macroscopically the homogeneous distribution of the NS in the polymer matrix in both cases. All the CGPEs were reticulated onto a glass-slide and then peeled off, being perfectly self-standing. The morphology of the different CGPEs was further studied by FESEM analysis both in top-view and cross-section modes. Pictures of the CGPE without NS (Figure 2d,g) demonstrate a globular structure, probably due to bubbles forming during the thermal treatment. Indeed, BMA possesses a low evaporation point and probably begins forming gases during the polymerization. Such a phenomenon did not seem to appear in the formulations containing NS; on the contrary, the surfaces of the CGPE containing 5 wt% (Figure 2e) and 10 wt% (Figure 2f) of NS were smooth and homogeneous. However, while the cross-section of the CGPE containing 5 wt% (Figure 2h) of NS appeared homogeneous and regular, the one of the CGPE containing 10 wt% (Figure 2i) presented a porous structure caused by structural inhomogeneities. The CGPEs’ thicknesses, as measured on cross-sections, were 25 µm without NS (Figure 2g), 40 µm with 5 wt% of NS (Figure 2h), and 85 µm with 10 wt% of NS (Figure 2i).

**Figure 3:**
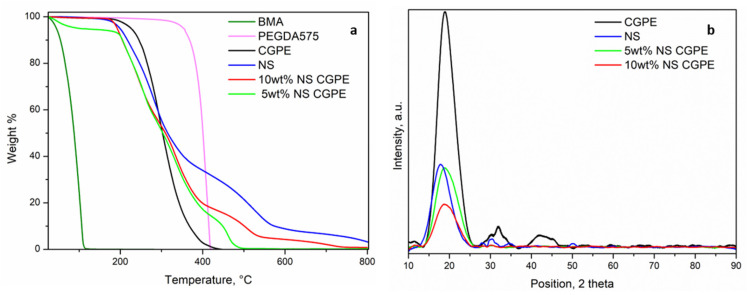
TGA traces of the CGPE precursors and the different CGPEs prepared (**a**). XRD patterns of NS, CGPE without NS, with 5 wt% NS, and 10 wt% NS (**b**).

The thermal stability of the CGPEs was investigated by TGA and compared to the analysis of each component of the precursor solution; results are reported in Figure 3. While the BMA monomer starts evaporating at around 100 °C (T_eb_ = 163 °C), PEGDA was thermally stable up to 400 °C. NS showed a more complicated profile with different weight losses (200 °C, 400 °C, and 600 °C) (see Table S1). The CGPE without NS started degrading around 230 °C, therefore between BMA and PEGDA, and without any intermediary plateau, thus demonstrating a complete polymerization. Both CGPEs containing NS started degrading a little earlier (around 200 °C), precisely following the degradation profile of pure NS. This phenomenon can be explained by their effect on the increase of amorphous fraction in the polymer matrix, as discussed in the following paragraph [25]. These results indicate that CGPEs containing NS can be used as a safe and reliable solid electrolyte separating the anode and cathode even at elevated temperatures, which could greatly improve the safety of the Li-O_2_ battery [26].

Crystalline phases in polymer electrolytes represent an obstacle to Li-ions conduction as they block the segmental motion of polymer chains. Therefore, XRD analysis was performed on the different CGPEs and on the NS additive to assess this characteristic (Figure 3b). The CGPE without additive showed a pronounced peak at 2θ = 20° and two smaller peaks at 30° and 45°, indicating a semi-crystalline nature. In particular, the shape of the first most intense peak reflects the ordered packing of polymer chains while the second and third peaks denote the ordering inside the main chains, at different ranges [27]. The NS powder showed a single peak around 2θ = 20°, which was therefore superposed to the one of the polymer matrix. This superposition does not allow us to draw any definitive conclusion regarding the presence of crystalline regions in the CGPEs containing NS additives. However, the intensity of the peak in the samples with 5 wt% and 10 wt% of NS, being lower than that of the polymer matrix without additive, suggests an overall decrease of crystallinity in the samples containing NS. In fact, the absence of crystalline phases, hinting to a highly amorphous matrix, has been demonstrated to not only be beneficial to ionic conductivity, as previously explained, but also to a more homogeneous Li plating [24].

Additionally to the crystalline nature of the polymer matrix, the liquid electrolyte uptake is another very important factor to ensure a good ionic conductivity inside the CGPE. The results obtained (see Table 1) show a much higher uptake using NS additives, demonstrating their good compatibility with the liquid electrolyte, in our case LiTFSI 0.5 M in DMSO. Moreover, as previously demonstrated, cross-linked polymer matrices containing additives show a better capability in retaining liquid electrolyte over time when compared to traditional glass fiber separators [28].

**Figure 4:**
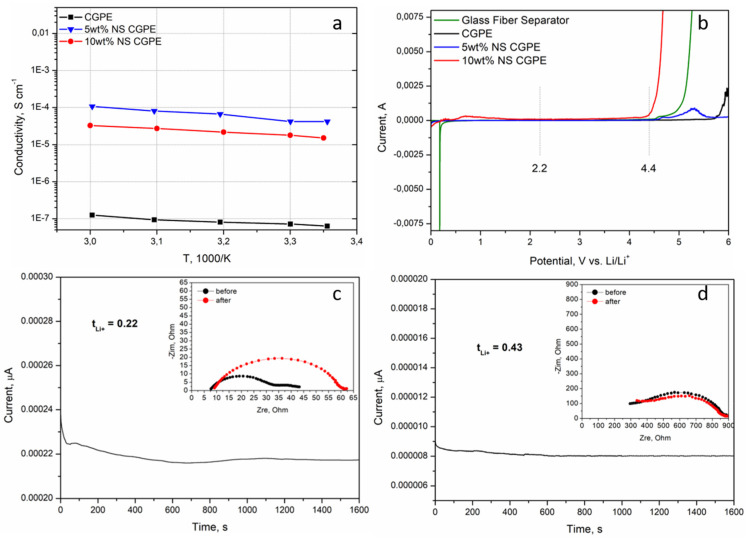
Ionic conductivity vs. temperature plot of the different CGPEs (**a**), LSV profile of the different CGPEs compared to a commercial glass fiber separator impregnated with liquid electrolyte (LiTFSI 0.5 M in DMSO) (**b**). Potentiostatic polarization analysis of a symmetric cell containing the glass fiber separator impregnated with the liquid electrolyte (**c**) and a symmetric cell containing the CGPE with 5 wt% NS (**d**).

In order to experimentally verify the influence of the two previously discussed parameters, ionic conductivities of the CGPE without NS and of the two CGPEs containing NS were assessed by EIS from room temperature to 60 °C. The results are reported in Figure 4a. In all cases, the conductivity increased with temperature, demonstrating that the increase of temperature leads to a faster movement of the polymer chains and, therefore, easier Li-ion transport [26]. The polymer matrix without additive had the lowest conductivity as could be predicted from the XRD (Figure 3b); indeed crystalline portions partially block the movement of polymer chains, hampering Li-ion transport. The highest ionic conductivity of the CGPE containing 5 wt% of NS can be attributed to two reasons. Firstly, BMA was cross-linked with PEGDA and modified with NS, which significantly reduced the crystallinity of the cross-linked polymer (Figure 3b), thus accelerating the transport of Li-ions inside the electrolyte [28]. Secondly, the as-modified CGPE was able to retain far more liquid electrolyte (Table 1), helping Li-ion conduction through a mixed mechanism between polymer chain motions and classic Li-ion diffusion in a liquid electrolyte.

The decomposition of the electrolyte at upper voltage is a serious issue affecting both the performance and the safety of the Li-O_2_ battery. Hence, an electrolyte with stable electrochemical window has great significance for the stable operation of such system [26]. The electrochemical windows of the CGPE without additive and of the CGPEs containing different NS contents were tested by LSV and compared to a glass fiber separator containing liquid electrolyte; the results are reported in Figure 4b. Interestingly, the CGPE without additive was the one demonstrating the largest stability window, up to 5.5 V. This can be explained by its semi-crystalline nature and low electrolyte uptake, both reducing the possibilities of side reactions with Li. Even if slightly narrower, the stable electrochemical window of the CGPE containing 5 wt% NS can ensure its integrity during battery operation for this specific application (namely between 2.25 V and 4.4 V). Given its limited stability window, the CGPE containing 10 wt% NS was not further considered in this work.

t_Li+_ is an important parameter for electrolytes, indeed the higher the t_Li+_, the better it can mitigate the anion accumulation around the electrode/electrolyte interface by alleviating the concentration polarization [29]. Indeed, such polarization causes concentration gradients at the electrode/electrolyte interface, which have been demonstrated in literature to be one of the principal causes of Li dendrite nucleation and growth, thus causing capacity fading and, more importantly, high instability and low safety of the cells [30,31]. The t_Li+_ value of the 5 wt% NS CGPE was assessed by a combination of potential polarization and EIS at room temperature (Figure 4d) and compared to the one of a commercial glass fiber separator impregnated with liquid electrolyte (Figure 4c). The results show that the t_Li+_ value of the 5 wt% NS CGPE was almost twice that of the liquid electrolyte on the glass fiber separator. This phenomenon is attributed to the synergy between the composite polymer matrix and the liquid electrolyte, which have a very positive effect on Li-ion migration. The compatibility of the 5 wt% NS CGPE with metallic Li negative electrode was enhanced; furthermore, the transition of the crystalline phase of the polymer matrix alone, leading to the increase of the amorphous region, greatly reduces the Li-ion migration resistance [26]. In addition, such t_Li+_ values are in line with previously reported ones for GPEs [11,28].

One important issue of the Li-O_2_ technology is the so-called O_2_ cross-over. In particular, O_2_ gets dissolved in the liquid electrolyte and comes into contact with the Li metal anode, thus causing its oxidation and successive passivation, leading to capacity fading and an eventual safety hazard. To assess the effect of NS additive on blocking O_2_, permeation measurements were performed on the CGPEs with and without NS. The results are reported in Table 2 and show an O_2_ permeation reduced by 80% in the membrane containing NS, indeed confirming the blocking role of NS and its benefit in enhancing cell safety.

**Figure 5:**
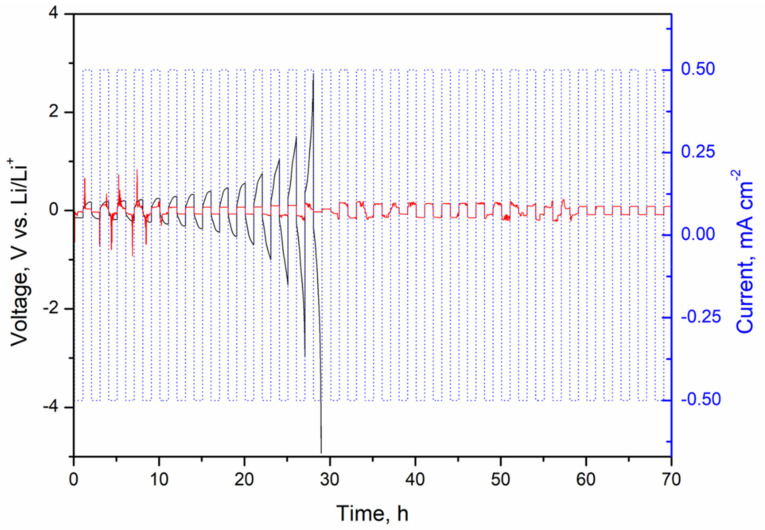
Voltage profile of the Li plating/stripping galvanostatic cycling at a current density of 0.5 mA cm^−2^ (specific capacity: 0.5 mAh cm^−2^) on symmetric Li|LE|Li cell (black) and Li|5 wt% NS CGPE|Li cell (red).

The interfacial stability of the electrolyte with Li anode was further elucidated by galvanostatic Li plating/stripping on symmetric Li|5 wt% NS CGPE|Li cells. For comparison, symmetric Li|LE|Li cells were simultaneously tested, where LE indicates the liquid electrolyte (LiTFSI 0.5 M in DMSO) in the commercial glass fiber separator, to assess LE compatibility with Li metal at different current densities. A first test was carried out at 0.3 mA cm^−2^ with a limited capacity of 0.3 mAh cm^−2^ on symmetric cells containing LE, CGPE without NS, and CGPE with 5 wt% NS, respectively. The first five cycles are reported in Figure S2 and show that, while the cell containing the LE and the one containing the CGPE with 5 wt% NS presented a stable profile with relatively low polarization, the cell containing the CGPE without NS presented severe fluctuations with a large voltage polarization, implying Li dendrite growth from the first cycles on the Li metal surface [26]. The test was repeated on cells containing the LE and the 5 wt% NS-based CGPE at a higher current density of 0.5 mA cm^−2^ and a limited capacity of 0.5 mAh cm^−2^; the obtained profiles are shown in Figure 5.

The symmetric cell containing the 5 wt% NS CGPE shows high overpotential in the first cycles, which is speculated to be attributed to the preconditioning of the cell [32]; then, the voltage feedback becomes smooth and stable at a relatively low polarization voltage, showing excellent cycling stability and demonstrating that the growth of Li dendrites can be inhibited to a certain extent. This improvement can be attributed to the stabilization of the electrolyte-electrode interface [11]. In contrast, the interfacial resistance of the liquid electrolyte-based cell kept increasing from the first cycles on, which indicates both the decomposition of liquid electrolyte and the constant formation of a SEI layer, and the accumulation of dead Li, up to the 14th cycle where the cell was failing [4,8]. The reason for the lower polarization of the cell containing the 5 wt% NS-based CGPE after 35 cycles compared to the one of the LE cell at the 14th cycle can be attributed to the improved t_Li+_ (see Figure 4c,d), contributing to a lowered concentration polarization in the 5 wt% NS-based CGPE [28]. This result demonstrates that high ionic conductivity and t_Li+_ improvement effectively stabilized the Li interface by promoting uniform Li plating/stripping and stable interfacial layers, hence enhancing the cell safety.

**Figure 6:**
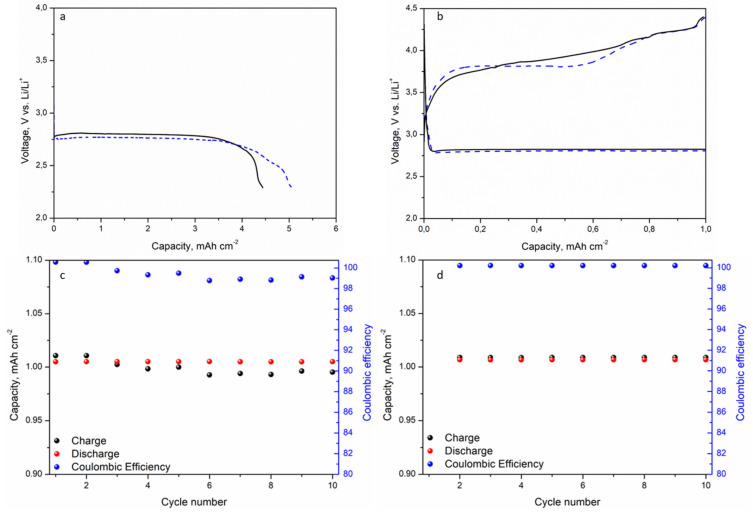
Full discharge profile of the STD cell (solid black line) and 5 wt% NS CGPE cell (dotted red line) (**a**). The 2nd cycle profile of the STD cell (solid black line) and 5 wt% NS CGPE cell (dotted red line) (**b**). Discharge/charge capacities and Coulombic efficiency vs. cycle number of the STD cell (**c**) and 5 wt% NS CGPE cell (**d**).

Galvanostatic discharge tests (Figure 6a), from OCV to 2.25 V vs. Li/Li^+^, were performed to evaluate the full discharge capacity of a cell containing the 5 wt% NS CGPE compared to a cell containing liquid electrolyte in a commercial glass fiber separator (referred to as STD), at a current intensity of 0.025 mA cm^−2^. The full cells were assembled using a simple, uncatalyzed, commercial GDL as a cathode. Prior to measurements, Li-O_2_ cells rested 6 h at OCV under O_2_ flow. Results show that the cell containing the 5 wt% NS CGPE was able to discharge for a longer time compared to the STD one, reaching an areal capacity as high as 5.05 mAh cm^−2^ in the first case, against 4.44 mAh cm^−2^ in the second one. As shown in Figure 6a, the potential of the STD cell abruptly plummeted at around 4.4 mAh cm^−2^, probably because of extended Li passivation due to O_2_ cross-over and irregular Li plating and striping. In particular, in literature, noticeable amounts of Li_2_CO_3_ have been associated to the oxidation of DMSO in long discharge conditions, and excess of O_2_, in particular at the anode side, has been considered a critical factor in driving such chemical and electrochemical side reactions [33,34]. Hence, in these conditions, both anode and electrolyte consumption could be responsible for the potential drop in the STD cell.

To investigate the cyclability of cells, galvanostatic cycling tests were carried out by limiting the initial full discharge capacity (based on the STD cell performance) to 20% and the potential to 2.25 V in discharge and 4.4 V (vs. Li/Li^+^) in charge, at the constant current density of 0.025 mA cm^−2^. During the tests, cells were continuously purged with dry O_2_ at a flow rate of 3.0 mL min^−1^. Prior to measurements, the Li-O_2_ cells rested 6 h at OCV under O_2_ flow.

Figure 6b reports the voltage vs. capacity plot of the second cycle in the STD cell (solid black line) and in the cell containing the 5 wt% NS CGPE (dotted red line). In the last one, the decrease of the charging voltage plateau indicates that lower amounts and more reversible side products were formed at the cathode during the previous discharge, thus allowing an easier conversion during the next recharge, which therefore took place at lower potentials. Such results support the assumption that DMSO decomposition occurs at both electrodes, but mainly at the Li/electrolyte interphase [35]. This seems to confirm that the 5 wt% NS CGPE can effectively relieve, to some extent, such side reactions. This fact is further verified looking at Figure 6c,d, respectively, reporting charge/discharge capacities and Coulombic efficiency for the STD cell and the cell containing the 5 wt% NS CGPE. Indeed, after the third cycle, in the STD cell, the charge capacity drops under the discharge capacity, meaning that the system was not able to fully re-convert the discharge products under the cut-off potential of 4.4 V, thus provoking an accumulation at the surface of the electrodes. Such a phenomenon was not verified in the cell containing the 5 wt% NS CGPE, where the discharge and charge capacity values were very stable over the first 10 cycles. Another important fact to keep in mind is that constant O_2_ flow leads to the intense evaporation of LE in the system [36]. Compared to LE, the 5 wt% NS CGPE almost doubles the t_Li+_ value and decreases the recharge potential, thus enhancing the cycle stability.

These observations agree with previous studies, showing that electrolytic properties in Li-O_2_ batteries, such as ionic conductivity and t_Li+_, play a significant role in Li-O_2_ battery behavior both in terms of cyclability and safety [11].

**Figure 7:**
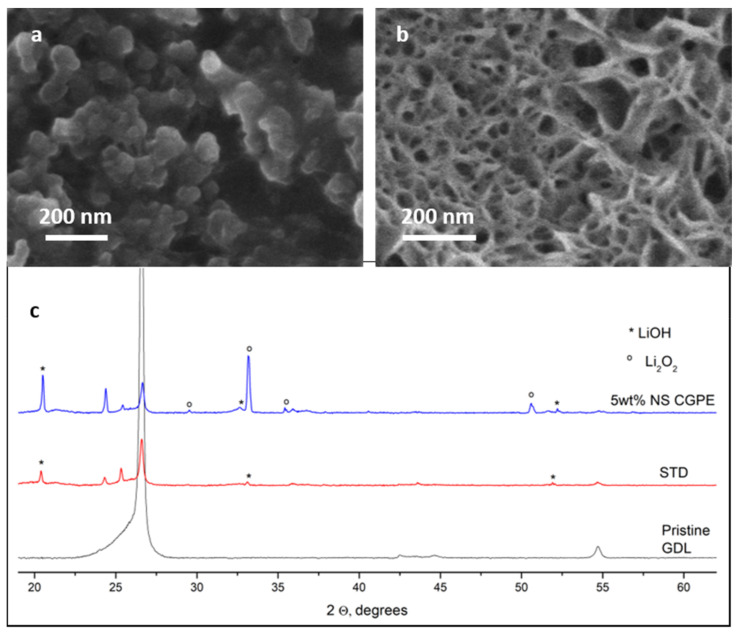
FESEM micrographs of the cathode surfaces after 10 cycles for the STD cell (**a**) and the 5 wt% NS CGPE cell (**b**). XRD patterns of a pristine cathode, the STD cathode and the 5 wt% NS CGPE cathode after 10 cycles (ICCD database: Li_2_O_2_ [00-009-0355] and LiOH [00-004-0708]) (**c**).

DMSO has been widely studied as an electrolyte solvent for Li-O_2_ cells and some papers report that it promotes the formation of flake-like agglomerates, apart from toroids, on the cathode surface. Such agglomerates are considered to be mixed LiOH and Li_2_O_2_ nanocrystallites [37] and they were particularly visible on the surface of the cathode cycled in the cell containing the 5 wt% NS CGPE (see Figure 7b). As a matter of fact, the surface of the STD cell cathode seemed to be covered by a mainly amorphous layer (see Figure 7a), while the deposit on the membrane cell cathode seemed much more crystalline (see Figure 7b). It is important to keep in mind that the cycling time used for this study was very long (50 h/cycle), thus allowing the degradation of some byproducts and the formation of amorphous species.

This result was confirmed by the XRD analysis of the same cathodes (see Figure 7c). Indeed, the spectrum of the cathode from the cell containing 5 wt% NS CGPE showed the typical peaks of crystalline Li_2_O_2_, while they could not be seen on the spectrum of the STD cell cathode. In particular, the diffraction peaks at 32.9° and 35.0° are attributed to the (100) and (101) crystal planes of Li_2_O_2_ [11,38].

Interestingly, crystalline LiOH could be found on both cathodes; this can be explained by the fact that, while the use of DMSO solvent in Li-O_2_ cells electrolytes can stabilize the soluble superoxide intermediates, such process is usually accompanied by side reactions, resulting in the formation of additional discharge byproducts aside from Li_2_O_2_ [39]. Possible reactions provoked by O_2_^−^ are illustrated in Equations (4) and (5) [37]:

CH_3_SOCH_3_ + O_2_^−^ → CH_3_SOCH_2_^−^ + O_2_H (4)

2 O_2_H + 2 Li^+^ + 2 e^−^ → 2 LiOH + O_2_ (5)

Comparing the morphology of both cathodes’ surfaces, we can conclude that the accumulation of mainly amorphous and poorly reversible side products onto the STD cell cathode surface provoked pore clogging, limiting O_2_ diffusion, and poor electronic contact between active material and carbon matrix, thus explaining poorer rechargeability. In contrast, crystalline deposits onto the 5 wt% NS CGPE cell cathode seemed to maintain the porosity of the cathode, allowing better oxygen flow and easier electron exchange. A hypothesis concerning morphology difference could regard the better O_2_ retention at the cathode surface, thanks to the blocking action of the 5 wt% NS CGPE, thus allowing better reaction kinetics and therefore better cyclability and higher safety.

## 4. Conclusions

In summary, we have successfully developed a CGPE composed of a highly cross-linked polymer matrix, encompassing NS, and activated with liquid electrolyte. The polymer matrix was easily obtained by thermally activated one pot free radical polymerization in bulk, directly dispersing NS into the precursor solution. We verified that the cross-linking of the polymer matrix enhanced its mechanical properties, thus efficiently reducing dendrites growth. Moreover, we demonstrated that the role of NS is twofold: first, it allows to limit the formation of crystalline domains; second, it blocks O_2_ permeation and cross-over to the anode and the consequent anode passivation. The successive activation of the polymer matrix with liquid electrolyte, coupled with the limitation of crystalline domains, ensure a good ionic conductivity as well as a doubled t_Li+_ value compared to liquid electrolyte on a commercial separator. The combination of these properties guaranteed a smooth Li plating and stripping at room temperature for more than 35 cycles at a current density as high as 0.5 mA cm^−2^. In the meantime, the standard cell, containing the liquid electrolyte with a commercial separator, experienced dangerous polarization and failed after only 14 cycles. In a full cell containing a simple, uncatalyzed, commercial GDL as a cathode, the cell assembled with the CGPE demonstrated a full capacity of 5.05 mAh cm^−2^ and a much higher stability upon cycling compared to the standard cell. These results demonstrate that the use of a specifically tailored CGPE could greatly enhance the safety as well as the performances of Li-O_2_ batteries, paving the way towards their practical applications.

## Figures and Tables

**Table 1:** LEU values of the different CGPEs.

Sample	CGPE	CGPE with 5 wt% NS	CGPE with 10 wt% NS
LEU	40%	110%	100%

**Table 2:** Summary of the permeating flow (J) and permeability coefficient (KP), obtained at 25 °C, 1 bar, and 0% of relative humidity for CGPE and 5 wt% NS CGPE.

Sample	J (cm^3^/(m^2^ 24 h))	KP (Barrer)	KP (g/(m^2^ 24 h))
CGPE	4720.115	0.030	5.99
5 wt% NS CGPE	799.711	0.006	1.02

## Data Availability

Additional data may be provided upon request to the corresponding author.

## Supplementary Materials

The following are available online at https://www.mdpi.com/article/10.3390/polym13101625/s1, Table S1: Summary of TGA analysis, Figure S1: FESEM micrograph of raw nanosponge aggregates, Figure S2: Voltage profile of the first 5 cycles of Li plating/stripping cycling at a current density of 0.3 mA cm^−2^ (specific capacity: 0.3 mAh cm^−2^) on symmetric Li|LE|Li cell (a), Li|CGPE|Li cell (b) and Li|5 wt% NS CGPE|Li cell (c).
