# Supplementary material for: Nanosponge-Based Composite Gel Polymer Electrolyte for Safer Li-O2 Batteries
Source: Polymers (Basel). 2021 May 17;13(10):1625. doi: 10.3390/polym13101625 (PMC8156716; doi:10.3390/polym13101625)
Supplement: Supplementary file 1 [file polymers-13-01625-s001.zip › polymers-1217591-supplementary.pdf]

# Supporting Information

# Nanosponge-based Composite Gel Polymer Electrolyte for Safer Li-$O_2$ Batteries –

**Julia Amici [1,*], Claudia Torchio [1], Daniele Versaci[1], Davide Dessantis [1], Andrea Marchisio [1], Fabrizio Caldera[2], Carlotta Francia[1] and Silvia Bodoardo [1]**

[1] Electrochemistry Group, DISAT, Politecnico di Torino, C.so D.ca degli Abruzzi 24 10128 Torino (TO) ITALY;
[2] Department of Chemistry, Università degli Studi di Torino, Via Pietro Giuria 7, 10125 Torino (TO), ITALY
* Correspondence: julia.amici@polito.it

**Table S1.** Summary of TGA analysis.

| Sample | $T_5$ (°C) | $T_{50}$ (°C) |
|---|---|---|
| BMA | 45 | 88 |
| PEGDA 575 | 354 | 401 |
| NS | 199 | 315 |
| CGPE | 226 | 303 |
| 5 wt% NS CGPE | 201 | 303 |
| 10 wt% NS CGPE | 192 | 310 |

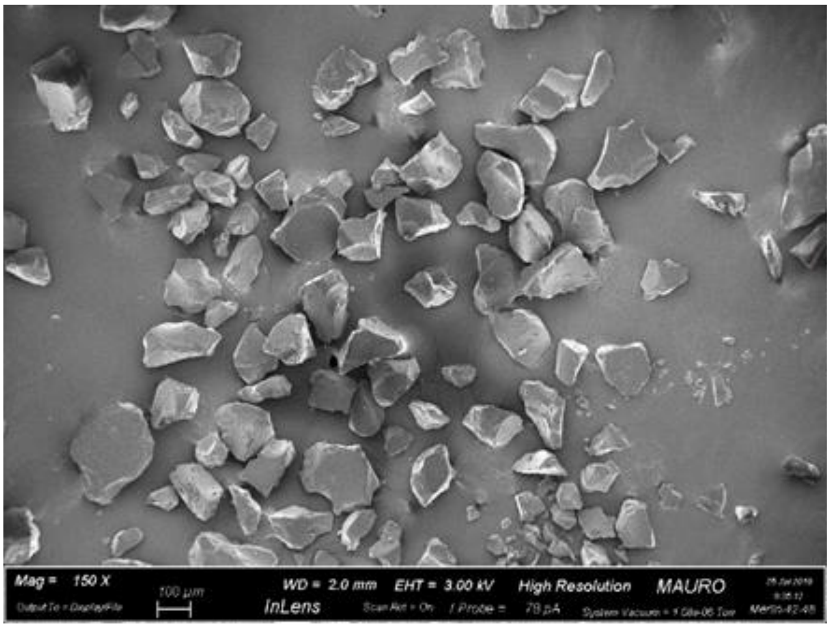


**Figure S1.** FESEM micrograph of raw nanosponge aggregates.

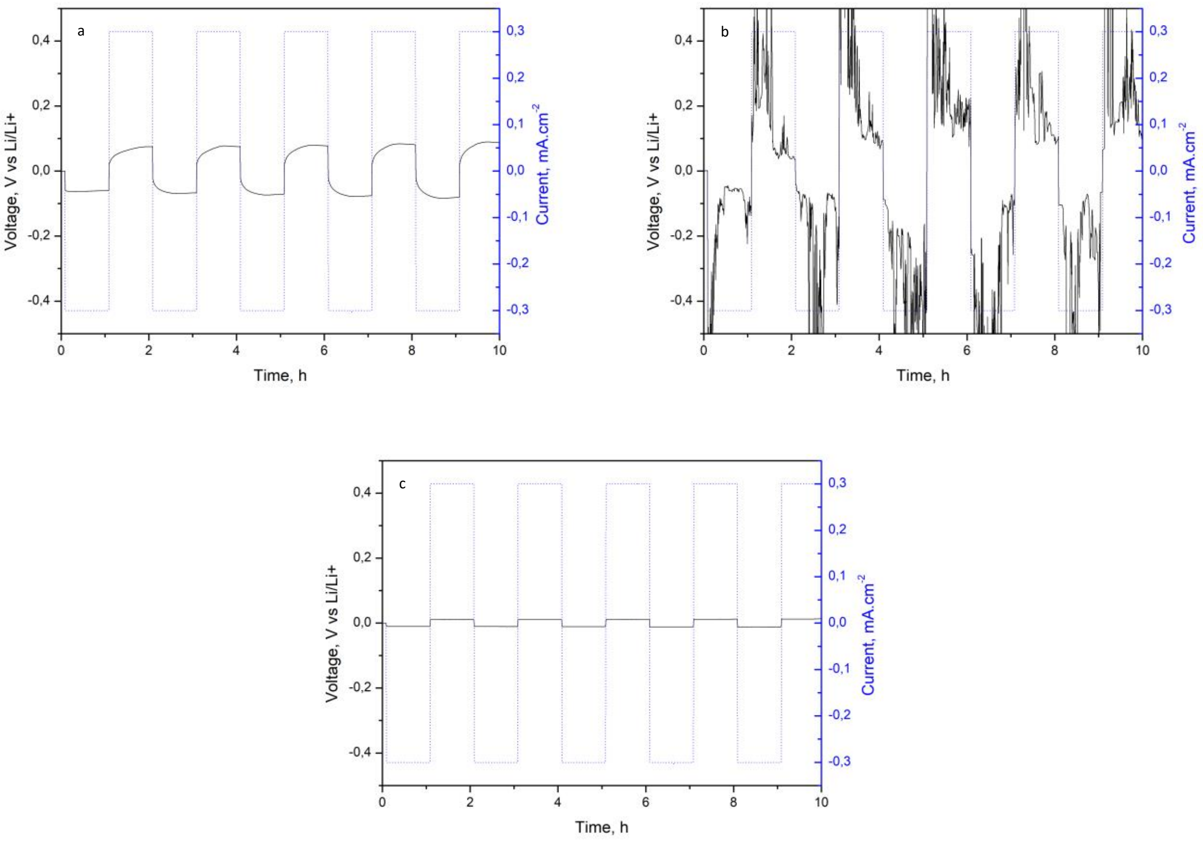


**Figure S2.** Voltage profile of the first 5 cycles of lithium plating/stripping cycling at current density of 0.3 mA·cm$^{-2}$ (specific capacity: 0.3 mAh.cm$^{-2}$) on symmetric Li|LE|Li cell (a), Li|CGPE|Li cell (b) and Li|5 wt% NS CGPE|Li cell (c).
